# Using Herbal dyes as an alternative staining method for sperm evaluation

**DOI:** 10.1002/vms3.268

**Published:** 2020-04-23

**Authors:** Mohammadreza Ebrahimi, Abbas Parham

**Affiliations:** ^1^ Division of Physiology Department of Basic Sciences Faculty of Veterinary Medicine Ferdowsi University of Mashhad Mashhad Iran; ^2^ Stem Cell Biology and Regenerative Medicine Research Group Institute of Biotechnology Ferdowsi University of Mashhad Mashhad Iran

**Keywords:** black mulberry, bull, henna, natural dye, safflower, sperm, staining

## Abstract

Staining, as a valuable method for sperm morphological assessment, has been used to determine sperm abnormalities, fertilization capability and sperm suitability during freezing‐thawing process. Synthetic dyes have been used for sperm viability and morphological evaluation. However, most of them have been made from chemical substances and have a perilous effect on the environment. In the current study, we evaluated three different natural dyes as the natural sources of dye for sperm staining. Bull frozen semen was used and prepared on slides for staining. Aqueous extract dye of black mulberry (BM), henna (HA), safflower (SA) and eosin‐nigrosine (control group) were used for sperm staining. Additionally, the effect of staining dyes on viability and some morphological parameters (head area: HR, head abnormality: HB and tail abnormality: TA) were evaluated. Although none of the natural dyes could detect viability of the sperm cells, safflower stain (HR: 26.55 µm, HB: 0% TA: 28%) and black mulberry stain (HR: 25.07 µm, HB: 2% TA: 3%) compared to control group (HR: 34.29 µm, HB: 4%, TA: 4%) provoked a strong reaction in the sperm cells, so that the sperms were observed yellow and red respectively. The reaction of sperm cells to the henna dye was very poor and it did not stain the sperm cells. Thus, the present study demonstrated that SA and BM dyes are able to stain the spermatozoa and with further modification could be used as alternative dyes for sperm staining in the study of sperm morphology, but not viability. Staining with these dyes can be an alternative to current costly chemical staining methods.

## Graphical abstract

In this study, we assessed the validity of some herbal dyes including black mulberry (BM), henna (HA) and safflower (SA) for sperm evaluation. Our results revealed that SA and BM dyes are able to stain the spermatozoa and could be used as alternative dyes for sperm staining in the study of sperm morphology.

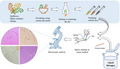

## 1 INTRODUCTION

Sperm morphology is an important criterion for evaluation of male fertility (Gosz, Mirny, Horbowy, & Zi˛etara, M. S., 2010; Lasiene et al., 2013) and correlates with fertilization and cryopreservation outcomes (Kalthur, Adiga, Upadhya, Rao, & Kumar, 2008). There are a variety of semen morphological evaluation tests including automated and standard methods. Computer‐assisted sperm analysis (CASA), as the only automatic morphology assessment system, is relatively expensive and time consuming compare to the manual analysis. Also, this method is not free of errors and require further developments to use in the sperm morphology assessments.

Thus, sperm manual examination and the WHO guidelines should serve as a reference and the gold standard for sperm evaluation (Talarczyk‐Desole et al., 2017). Due to this, numerous cell staining methods.

are available for assessment of viability and morphology of sperm. Eosin and nigrosin (Kondracki, Banaszewska, Wysokińska, & Iwanina, 2012), silver nitrate staining (Andraszek, Banaszewska, Czubaszek, Wójcik, & Szostek, 2014) and Giemsa and Diff‐Quick (Kruger et al., 1996) are common standard dyes that allow the evaluation of the sperm structure and/or viability. However, most of them are synthetic and not biodegradable substances that can be hazardous to environment and water sources (Bordoloi, Jaiswal, Siddiqui, & Tandon, 2017; Titford, 1996).

Alternatively, natural dyes, derived from plants, insects/animals and minerals are eco‐friendly and sustainable sources with minimum environmental impact (Kadolph, 2008). Regardless of their sources (plants, animals and minerals), they have been used for staining wool, skin, silk, carpet and cotton from many years ago (Jacobson & Wasileski, 1994; Watson, 1991). Due to these characteristics, there have been several scientific works that have revealed potential of natural dyes for histological studies, such as Hibiscus sabdariffa for the study of sperm morphology (Bassey, Osinubi, & Oremosu, 2012), black mulberry fruits (Morus nigra) for the identification and differentiation of parasites (Tousson & Al‐Behbehani, 2011) and nervous tissues (Tousson & Al‐Behbehani, 2010) and Lawsonia inermis (Henna) for staining the brain tissues (Alawa, Gideon, Adetiba, & Alawa, 2015). Generaly, each dyes may have a different effect on the staining of cell structures as a result of a different response to chemicals components. For example, Lącka, Kondracki, Iwanina, and Wysokińska (2016) and Maree, Plessis, Menkveld, and Horst (2010), indicated that dyes may influence the sperm morphology such as the form of sperm head and the final results could be different and species‐dependent.

Regarding these conceivable effects of dyes on sperm cells, we aimed to investigate and compare the potential of some native natural dyes that have not been examined in the study of bull sperm viability and morphology including safflower (*Carthamus tinctorius*), black mulberry (*Morus nigra*) and henna (*Lawsonia inermis*) so far.

## 2 MATERIAL AND METHODS

All experimental procedures were approved by The Ethics Committee of the Ferdowsi University of Mashhad, Mashhad, Iran.

### 2.1 Preparation of extract

Safflower (SA), black mulberry (BM) and henna (HA) were purchased from medicinal plant store and approved by the herbarium section (Mashad, Iran). In order to prepare the aqueous extracts, the plant materials were washed with sterile water, dried in an oven (60°C) for 24 hr and ground using mortar and pestle. The dye extraction was performed as previously described, with some modifications. Brifly, for extraction of BM (Tousson & Al‐Behbehani, 2011), HA (Udeani, 2015) and SA dyes (Shin & Yoo, 2012), approximately, 50 g of the dried plants were mixed with 100 ml of distilled water and heated to just below boiling point and immersed until the colour has been transferred from the dye solution. Then, concentrated juices were cooled. After cooling, two steps filtration process was used twice for extract purification. Initially, the extracts were centrifuged at 4,200 *g* for 30 min. The supernatants were collected and filtered through Whatman filter paper (no: 1). subsequently, they were dried in oven at 60°C. The residue was scraped, ground and stored under refrigeration (4–8°C) and dark condition to protect from light and thermal destruction (Figure S1).

### 2.2 Semen samples

Frozen Holstein bull semen (0.5‐ml straws) stored in liquid nitrogen were thawed in the warm water (37°C ‐ 30 s) and diluted with an equal volume of normal saline. Frozen semen from three bulls were used. Five straws from each bull were applied for each dye and five slides were stained for each straw.

### 2.3 Preparation of staining solution and staining method

One gram of the dried aqueous extract of safflower (SA), black mulberry (BM) and henna (HA) were dissolved in 2 ml of distilled water and stored at 4°C. For all groups, dye preparation and sperm staining was done at the same day. Sperm staining was done by smear method, using frozen‐thawed bull sperm and prepared herbal dyes from an aqueous extract of SA, HA and BM. Briefly, a drop of thawed and diluted semen was placed on a warmed slide and mixed with the double volume of the prepared herbal dyes in experimental groups or Eosin/Nigrosine (Carl Roth Gmbh + Co. KG, Germany) in the control group. Then, the drops were spread on a glass slide and were allowed to be air dried. To obtain a good contrast, the slides were slowly dipped into distilled water to remove debris.

### 2.4 Microscopic analysis

In each slide, sperms were evaluated at 1000X magnification under the Nikon E‐50i microscope and digital camera‐captured images. Subsequently, the morphometric measurements were done on the heads of 100 randomly selected sperms that were clearly visible in the microscope field of view. At the end, to assess the staining ability of the dyes, the brightness and the contrast of images were modified by Adobe Photoshop software and categorized as good, very good, excellent and bad (described by + and ‐, Table 1).

**Table 1:** The percentage of the tail and the head abnormality of the sperm cells (mean ± *SD*) and powers of different stains

	Safflower	Black mulberry	Henna	Eosin‐Nigrosin
Power of stain	+	++	−	+++
Tail abnormality (%)	28 ± 4.2^a^	3 ± 1.1^b^	4 ± 0.9^b^	4 ± 1.3^b^
Head abnormality (%)	5 ± 1.4^a^	9 ± 0.7^b^	3 ± 1.2^a^	4 ± 1.1^a^

Note

Different superscripts differ significantly in the same row.

Power of stain: −negative, + weak, ++ moderate, +++ strong

In each group, morphometric parameters including the tail and the head abnormality of the 100 randomly selected sperm (totally 500 sperms) were visually assessed (Barth & Oko, 1989) and presented in percentage (Table 1). To avoid bias in the evaluation, all slides were evaluated by three expert observers and analysis was done using the mean value of scores.

To assessing the effects of the natural dyes on the sperm size, 20 head areas of spermatozoa in each slide (totally 100 sperm head in each group) were measured by image analysis software (Image‐J). The head area data were stored in a database and exported for further statistical analysis. Statistical differences between the samples were tested using ANOVA and Tukey as post‐hoc test (STATISTICA version 10.0, StatSoft Inc., PL). Values of *p* ≤ .05 were considered statistically significant.

## 3 RESULTS

**Figure 1:**
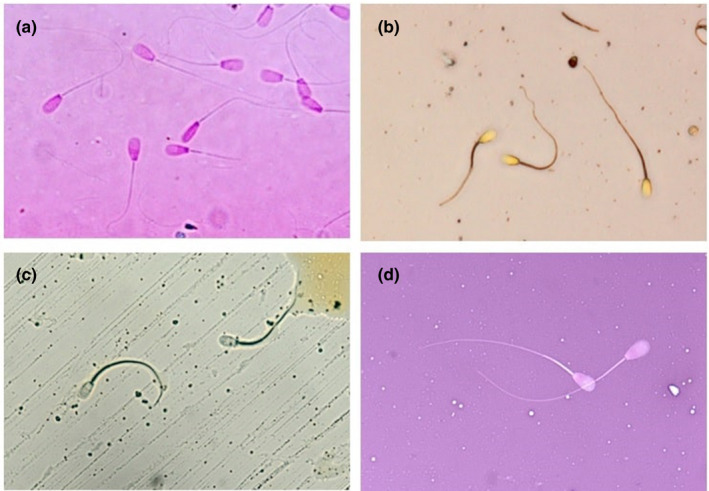
Results of bovine sperm staining with different dyes. (a) Black mulberry dye completely stained sperm cells with a little shrinking in the head, (b) Safflower dye stained all parts of the sperm cells, (c) Henna dye did not stain the bovine sperm cells and (d) Eosin/Nigrosin staining

As shown in Figure 1, the aqueous extract dye of safflower and black mulberry caused a strong reaction in the sperm cells and provide sharp contrast. The sperm cells were stained with safflower dye in yellow color, especially in the head and the middle part of the sperm cells which clearly increased the contrast between the light background and sperm cells. The acrosomal part of the head was lighter, however it was not distinct enough for precise measurement of the area and extent of the acrosome. Nonetheless, the outline of the head was sufficiently clear to identify and measure.

The sperm cells stained by black mulberry were observed in red color with pale pink background which made sperm easier to visualize. Additionally, the reaction of the sperm cells to the henna dye was very poor and it did not stain the sperm cells. Although the sperm cells stained with Eosin/Nigrosin in the control group had more transparency and provided the possibility to detect the live and the dead cells, none of the natural dyes were able to detect the live sperm cells.

**Figure 2:**
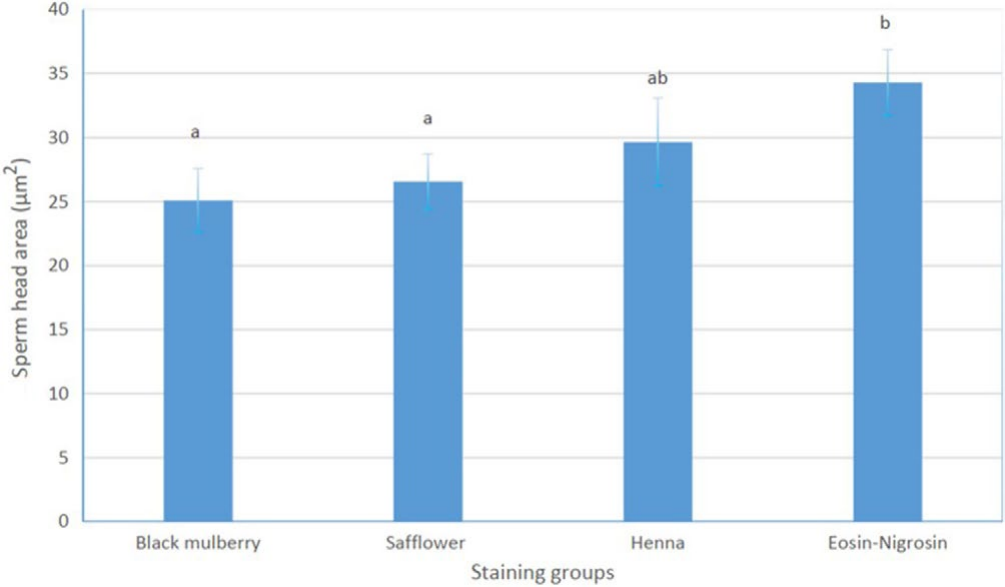
The effect of natural dyes on the head area of bovine sperm cells. Significant differences were presented by different letters

To evaluate the effects of natural dyes on morphology of sperm cells, the size of the sperm head area, as one of the sperm morphological parameters, was measured (Figure 2). The analysis of the data revealed that there were significant differences between the head area of the sperms stained by Eosin/Nnigrosin and natural dyes (*p* ≤ .05). Especially sperms stained with the BM and SA caused the sperm cell heads to shrink, and therefore, affected the size of the sperm head compared to those stained with Eosin/Nigrosin dye. Moreover, the effect of HA dye was lesser on the sperm head area compared to the control group.

Also, microscopic analysis of stained smears was done to compare effects of natural dyes on head and tail normal morphology (Table 1). The stained spermatozoa with HA showed a uniform morphology, while BM and SA indicated some abnormality in the head and tail, respectively.

## 4 DISCUSSION

The data presented here demonstrate that BM and SA are able to stain the spermatozoa. However, because of some undesirable defects on sperm morphology, they need to be optimized for staining and morphological assessment. Natural dye has its own characteristics and some of them may not be suitable for sperm staining. They may alter sperm dimensions depending on chemical characteristics and staining techniques, which had been proven in human and pig semen (Kondracki, Wysokińska, Kania, & Górski, 2017 and Maree et al., 2010). Some of these staining reagents have been found to affect the sperm size (Sancho, Pérez‐Sánchez, Tablado, Monserrat, & Soler, 1998). They cause sperm cell heads to swell (Kruger et al., 1987) or shrink (Gao et al., 1993).

The current study showed that sperm staining with BM dye has a great result in comparison with SA and HA dyes, while it is not as good as the result of the control group that showed a high contrast as well as the ability to detect the sperm viability. The result of BM staining was consistent with the result of the study conducted by Tousson and Al‐Behbehani (2010), which had reported that the extracted dye from black mulberry can be used as an alternative dye for identification and differentiation of different brain parts and nervous tissue cells. SA and BM dye stained all the sperm cells yellowish and reddish color (both live and dead cells) respectively, which improves the visibility of the sperm cells. However, the SA extracted dye stained the sperm heads not stronger than the control group and BM group.

The ability of a dye to stain cells or tissue structures is based on chemical properties. The pH of the aqueous extract of the BM is 3.85 making it an acidic dye (Ercisli & Orhan, 2008). Acidic dyes stain basic parts (cytoplasm) while the basic dyes stain the acidic parts (nucleus) (Avwioro, Aloamaka, Ojianya, Oduola, & Ekpo, 2005). In addition, these natural dyes contain hydroxyl or phenolic group that are responsible for the coloring ability (Chen et al., 2006). For example, quinonoids and anthocyanins are responsible for the color of Safflower (Yusuf, Shabbir, & Mohammad, 2017) and Black Mulberry (Kamiloglu, Serali, Unal, & Capanoglu, 2013 and Aramwit, Bang, & Srichana, 2010), respectively.

Based on these findings, the aqueous extracted dyes from SA and BM that stained the cytoplasm of the sperm cells—the basic part of the cells—may have acidic properties. Although HA extracted dye, in the control and the other groups, contains hydroxyl or phenolic compounds and is able to bind to basic elements of tissues via ionic bonding (Alawa et al., 2015), in our study, HA dye could not stain any part of the sperm cells that was in consistent with the findings of the study done by Deepali, Lalita, Deepika, Stem, and Hibiscus (2014) who had reported that henna dye has a low capacity to stain the fungal specimen due to their composition of pigments. However, there are several studies that demonstrated that henna is an effective dye for staining the different animal tissues without morphological changes (Alawa et al., 2015). These conflicting results may be related to the fact that the sperm membrane did not allow henna stain to pass through the sperm membrane or it does not have affinity to cytoplasm contents, unlike the other groups. It may be the result of difference in the structural composition of the dyes or staining mechanisms by which the dyes work.

The morphological analysis reveals that the two factors, that is, sperm head dimensions and shape can be affected by BM and SA dyes component, which leads to head shrinkage. Therefore, it may has a negative effect on the normal morphology of spermatozoon structures. However, other studies that have used BM as a natural dye to stain nervous tissues, did not report any negative result in the tissue structure (Tousson & Al‐Behbehani, 2011). Here we suspect that osmotic condition of BM and SA dyes could be the probable reason for shrinking the head of spermatozoa. Previous studies indicated that each staining technique use different chemical reagents which can cause either the sperm cell to swell or shrink by penetrating its membrane and influencing the osmotic balance (Maree et al., 2010 and Hidalgo, Rodriguez, & Dorado, 2007). Also bent tails were the most detected tail abnormality in SA staining which may be associated with low osmolality (Johnson, Jacobs, & Walker, 1991).

Moreover, these results might have obtained due to several other reasons including different characteristics of sperm as a single cell in comparison with that of nervous tissues as dense cell masses, the difference between the preparation of sperm sample compared with that of fixed tissue, which is a multi‐step process that may affect, and finally, according to the study by Almadaly et al., the proportion of dye concentration and the exposure time of the spermatozoa to the dye, which may affect the sperm head shape (Almadaly, Farrag, Shukry, & Murase, 2014).

Based on our knowledge, the present study is the first study that investigated the potential of BM, SA and HA extracted dyes for sperm staining. Despite of some undesirable effects, these natural dyes (BM and SA) have a good potential to be optimized and used as an alternative dye for sperm and histological staining and also for clinical pathology. They could be a promising alternative for conventional biological synthetic dyes that not only decreases the experimental costs but also reduces the environmental pollution due to being free of carcinogenic azo compounds.

## CONFLICT OF INTEREST

None of the authors have any conflict of interest to declare.

## AUTHOR CONTRIBUTION

**Mohammadreza Ebrahimi:** Conceptualization; Data curation; Formal analysis; Investigation; Methodology; Writing‐original draft. **Abbas Parham:** Conceptualization; Funding acquisition; Supervision; Writing‐review & editing. 

## Supporting information

Figure S1: Click here for additional data file.
