# Supplementary figures and images for: Using Herbal dyes as an alternative staining method for sperm evaluation
Source: Vet Med Sci. 2020 Apr 23;6(3):441–6. doi: 10.1002/vms3.268 (PMC7397902; doi:10.1002/vms3.268)

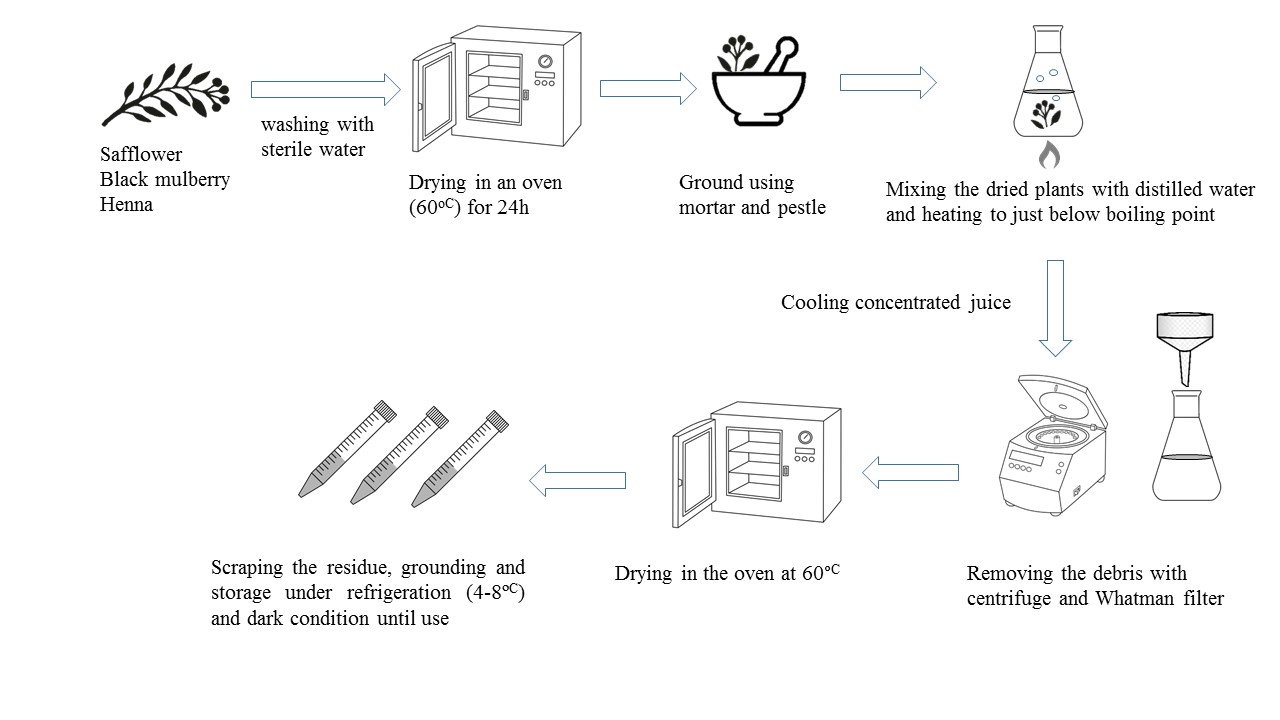

Supplement: Supplementary file 1 — Figure S1: [file VMS3-6-441-s001.jpg]
